# Durable complete response in leptomeningeal disease of EGFR mutated non-small cell lung cancer to amivantamab, an EGFR-MET receptor bispecific antibody, after progressing on osimertinib

**DOI:** 10.18632/oncotarget.28730

**Published:** 2025-05-29

**Authors:** Jinah Kim, Liam Il-Young Chung, Horyun Choi, Yoonhee Choi, Maria Jose Aguilera Chuchuca, Youjin Oh, Young Kwang Chae

**Affiliations:** ^1^University of Vermont Medical Center, Burlington, VT 05401, USA; ^2^Feinberg School of Medicine, Northwestern University, Chicago, IL 60611, USA; ^3^The Queen’s Medical Center, Honolulu, HI 96813, USA; ^4^NIH, Bethesda, MD 20894, USA; ^5^John H. Stroger, Jr. Hospital of Cook County, Chicago, IL 60612, USA

**Keywords:** amivantamab, monotherapy, rare EGFR mutation, NSCLC, leptomeningeal disease

## Abstract

Patients with NSCLC harboring uncommon EGFR mutations have limited therapeutic options and often poor outcomes, especially following progression on standard EGFR tyrosine kinase inhibitors (TKIs). Amivantamab, an anti-EGFR/MET bispecific antibody, shows efficacy in EGFR-mutated NSCLC, but its role in rare EGFR alterations and CNS involvement, including leptomeningeal disease (LMD), remains insufficiently characterized. We report a 67-year-old male with stage IVB NSCLC harboring EGFR G719A and A289V mutations who developed LMD while on osimertinib. After progression on immunotherapy and chemotherapy, amivantamab monotherapy was initiated. Treatment produced a durable response over 19 months, including a 32.2% reduction in tumor size at six weeks, and complete resolution of brain metastases and LMD by six months. Circulating tumor DNA analyses showed EGFR variant allele fractions decreasing from 25.6% to undetectable. This case challenges current paradigms, demonstrating that amivantamab monotherapy can effectively target rare EGFR mutations, achieve durable extracranial and CNS disease control, and potentially overcome blood-brain barrier limitations. These findings suggest that amivantamab’s utility may extend beyond established combination regimens and well-characterized EGFR mutations, offering an effective option for patients with atypical molecular profiles who lack standard therapies. Amivantamab monotherapy may represent a viable strategy for patients with uncommon EGFR mutations and CNS involvement, including LMD. Further research is warranted to elucidate underlying mechanisms, refine treatment strategies, and assess amivantamab’s broader applicability in similarly challenging NSCLC scenarios.

## INTRODUCTION

Patients with rare epithelial growth factor receptor (EGFR) mutations have limited treatment options and often face a poor prognosis due to the reduced efficacy of tyrosine kinase inhibitors (TKIs), which are the standard treatment for Non-small cell lung cancer (NSCLC). Amivantamab is an anti-EGFR/MET bispecific antibody with immune cell-directing activity. By inhibiting ligand binding and preventing receptor dimerization, it disrupts downstream signaling pathways that are vital for tumor cell growth and survival, thereby enhancing its antitumor effects [1]. In 2024, the FDA approved amivantamab for multiple indications in NSCLC: in combination with carboplatin and pemetrexed for EGFR exon 19 deletions or exon 21 L858R substitution mutations post-EGFR TKI treatment (September); with lazertinib as a first-line treatment for EGFR exon 19 deletions or exon 21 L858R mutations (August); and with carboplatin and pemetrexed as a first-line treatment for EGFR exon 20 insertion mutations (March) [2–4].

Here, we report a case that demonstrates a durable response to amivantamab monotherapy in a patient with uncommon EGFR mutations (G719A, A289V) in NSCLC, who developed leptomeningeal disease (LMD) while on osimertinib.

## CASE PRESENTATION

**Figure 1:**
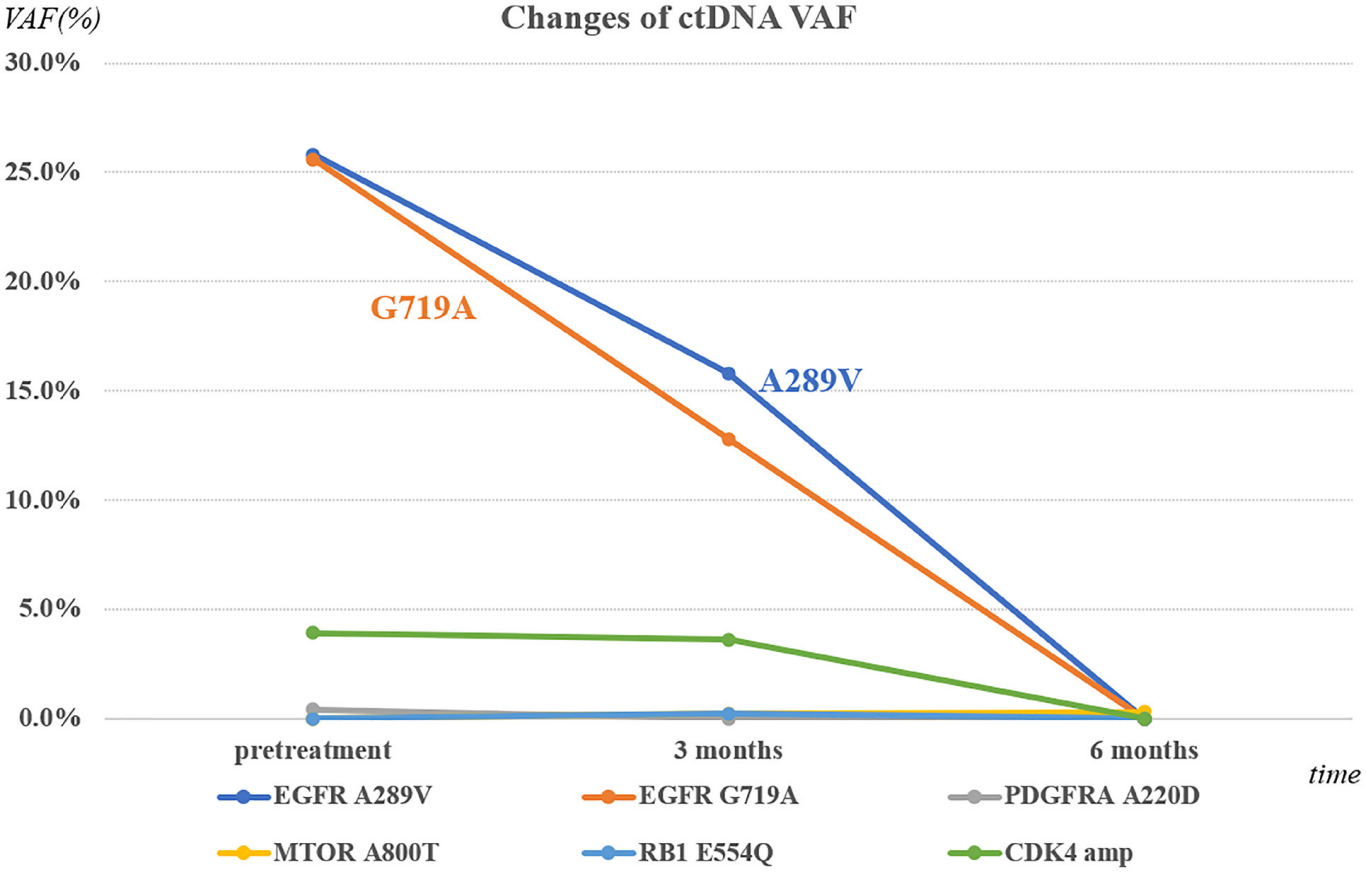
Change in variant allele fraction (VAF) by Next-generation sequencing (NGS) of circulating tumor DNA (ctDNA).

A 67-year-old Asian male with no smoking history presented with stage IVB squamous cell carcinoma (SCC) of the lung. A Computed tomography (CT) chest showed a spiculated 7.7 cm lesion in the right upper lung, along with hilar and subcarinal lymphadenopathy. A Magnetic resonance imaging (MRI) of the spine revealed multiple osseous metastases from the C6 to T12 vertebrae with pathologic compression fractures, but no central nerve system (CNS) involvement was observed. A biopsy of the primary lung mass confirmed poorly differentiated SCC with a programmed cell death ligand 1 (PD-L1) immunohistochemistry score of 70%. Next-generation sequencing (NGS) of circulating tumor DNA (ctDNA) was performed using the commercially available Guardantb360CDx (Guardant Health, Inc., Redwood, CA, USA) to evaluate up to 83 cancer-related genes. The NGS testing was conducted as part of standard clinical care in a CLIA-certified and College of American Pathologists accredited laboratory. Peripheral blood samples were collected in two 10 mL Streck tubes, and plasma was processed to assess single-nucleotide variants (SNVs), insertions, deletions, gene fusions or rearrangements, and copy number variants (CNVs). Mutations were annotated using OncoKB to define pathogenic variants. This analysis identified two potentially actionable mutations: EGFR G719A (exon 18, substitution) and EGFR A289V (exon 7, substitution) (Figure 1). The variant allele fractions (VAFs), representing ctDNA yield for EGFR A289V and EGFR G719A were 25.8% and 25.6%, respectively. Tissue NGS using Tempus xT (Tempus Inc, Chicago, IL, USA) revealed EGFR G719A and EGFR A289V with VAFs of 79.6% and 78.5%, respectively, and no other genetic alterations detected.

Osimertinib, a third-generation TKI, was initiated at 80 mg daily and continued until the patient developed pneumonitis alongside disease progression. After resolution of the pneumonitis, two cycles of carboplatin (504 mg IV) and paclitaxel (200 mg/m²) were administered, followed by the initiation of ipilimumab (1 mg/kg) and nivolumab (360 mg IV) the following week. Follow-up CT scans taken a week after starting immunotherapy and three months after discontinuing osimertinib revealed disease progression. CT and MRI scans showed a slight increase in the size of paratracheal and subcarinal lymph nodes, as well as subtle progression of multiple osseous metastases throughout the spine. The primary lung lesion was slightly smaller. An MRI of the brain identified at least six new parenchymal lesions, up to 9 mm in size, and leptomeningeal enhancement.

Consequently, immunotherapy was discontinued, and amivantamab monotherapy was experimentally initiated due to the patient’s borderline performance status, rendering him unsuitable for further chemotherapy or combination treatments. The initial amivantamab loading dose were 350 mg and 700 mg, followed by 1050 mg weekly. At this time, the patient was confined to a wheelchair and required substantial assistance with activities of daily living (ADLs) before starting amivantamab.

**Figure 2:**
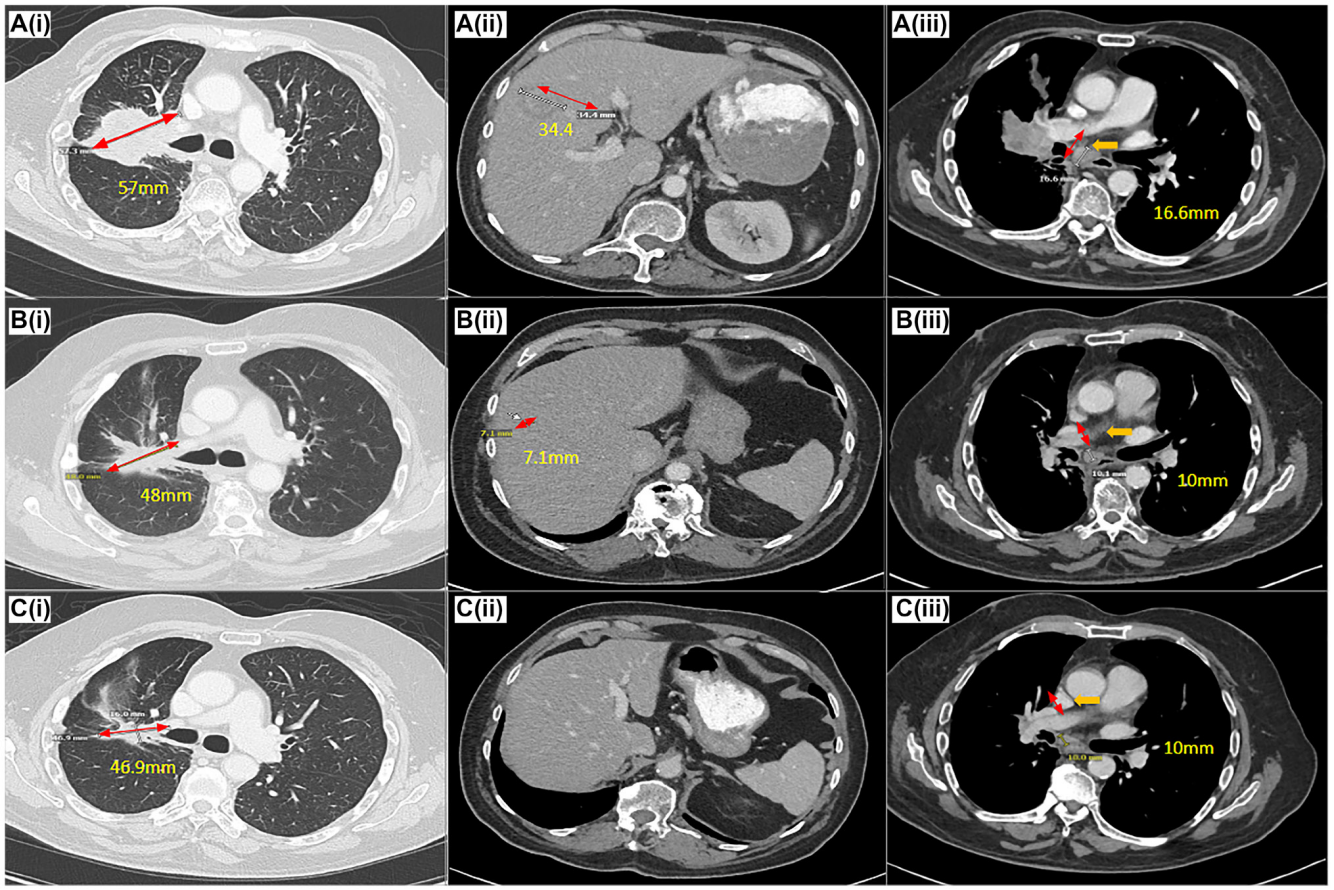
Target response on amivantamab based on RECIST 1.1 on CT scan. CT Chest (**i**), Abdomen/Pelvis (**ii**)(**iii**) at the time of pre-treatment (**A**), 6 months (**B**) (complete resolution of CNS disease), 19 months (**C**) demonstrating a decrease in size of the patient’s lung, liver, mediastinal lesions after treatment with amivantamab. Pre-treatment CT chest with a right upper lobe/perihilar mass, measuring 57 mm in the long axis (A(i)), decreased to 48 mm (B(i)) and 46.9 mm (C(i)) on post-treatment scans. Pre-treatment CT abdomen with a lobular liver lesion, measuring 34.4 mm in the long axis (A(ii)), decreased to 7.1 mm (B(ii)) and complete resolution (C(iii)) on post-treatment scans.

**Figure 3:**
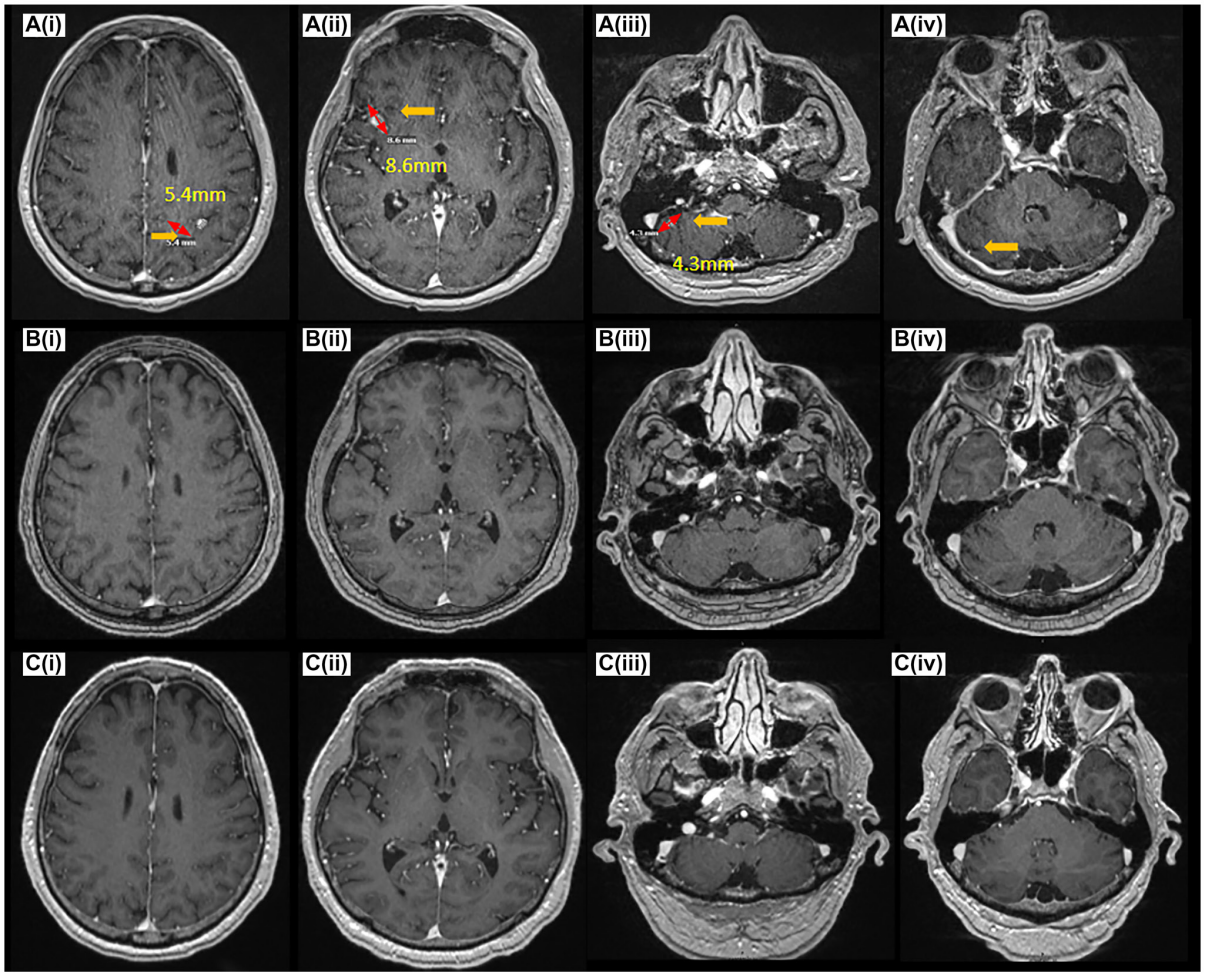
CNS disease response on amivantamab. MRI Brain at the time of pre-treatment (**A**(**i**–**iv**)), 6 months (**B**(**i**–**iv**)), 19 months (**C**(**i**–**iv**)) post-treatment demonstrating complete resolution of CNS disease at 6 months (B(i–iv)), and 19 months (C(i–iv)).

The patient tolerated amivantamab at 1050 mg IV weekly, then every two weeks starting from week 5, without major complications. Follow-up scans six weeks later showed a partial response with a 32.2% decrease in tumor size according to the Response Evaluation Criteria in Solid Tumors, version 1.1 (RECIST 1.1). An MRI of the brain demonstrated a reduction in the size of the parenchymal lesions and decreased leptomeningeal enhancement in the right Sylvian fissure. Repeat ctDNA NGS with Guardant 360 CDx also showed a marked reduction in the highest VAF from 25.6% (EGFR G719A) to non-detectable levels (Figure 1). Six months after starting amivantamab, there was complete resolution of CNS disease, including leptomeningeal enhancement. Evaluation of circulating tumor cells in the cerebrospinal fluid (CSF) found no evidence of their presence. The patient regained the ability to walk and perform ADLs independently. He has now been receiving amivantamab for 4 months, and his most recent CT scan continues to show stable disease in the primary lung lesion with no evidence of CNS involvement (Figures 2 and 3).

## DISCUSSION

This case highlights three unusual aspects of amivantamab: its activity against rare mutations, its efficacy as a monotherapy, and its effectiveness in treating CNS lesions including LMD.

First, this case involved a rare EGFR mutation. Exon 19 deletions are the most common EGFR mutations in NSCLC, accounting for approximately 48.5% of cases, followed by Exon 21 L858R mutations at about 34.0%. Exon 18 G719X point mutations which comprise 0.9–4.8% of all EGFR mutations are the third most common. Exon 20 insertions are notable, comprising 0.8–4.2% of mutations [5–10]. The National Comprehensive Cancer Network guidelines list afatinib and osimertinib as treatment options for certain uncommon EGFR mutations, including G719X, S768I, and L861Q [8]. Studies have shown that afatinib has a higher objective response rate (ORR) compared to first-generation TKIs for treating non-resistant uncommon EGFR mutations like G719X, with ORRs reaching approximately 60% [11–13].

Although amivantamab was not evaluated as a monotherapy in these studies, the CHRYSALIS-2 trial (Cohort C) assessed a combination of amivantamab and lazertinib for atypical EGFR mutations, including S768I, L861Q, and G719X. Results indicated positive outcomes, especially in treatment-naive patients, with an ORR of 51–55% and a median progression-free survival (mPFS) of 19.5 months [14].

The A289V mutation in EGFR is extremely rare in NSCLC [15]. This is the first reported case of NSCLC harboring both EGFR G719A and A289V mutations, although it has been noted that the G719X mutation can co-occur with other EGFR mutations like S768I [16]. Notably, the patient exhibited a similar pattern of VAF reduction for G719A and A289V, from 25.6% and 25.8% to non-detectable levels. This could indicate that the tumor cells in our case may share cellular properties that impact their response to amivantamab, suggesting that tumor heterogeneity could play a role in efficacy. This observation suggests that amivantamab may effectively reduce the tumor mutational burden of EGFR, not only in currently indicated mutations but also in other atypical types of mutations. In a real-world multicenter study, amavantamab has demonstrated strong efficacy in NSCLC patients with atypical EGFR mutations like G719X, S768I and L861Q with an 85.7% clinical response rate and a 100% disease control rate [17].

Secondly, this case demonstrated the efficacy of amivantamab as a monotherapy in rare EGFR-mutated NSCLC. Due to its limited efficacy and safety concerns, amivantamab has primarily been studied in combination with TKIs or chemotherapy. The phase III MARIPOSA trial (NCT04487080) showed that combining amivantamab with lazertinib reduced the risk of progression or death by 30% compared to osimertinib, with a mPFS of 23.7 months versus 16.6 months for those treated with osimertinib [18]. The phase III PAPILLON trial (NCT04538664) is assessing the efficacy of amivantamab in combination with chemotherapy compared to chemotherapy alone, while the phase I/II METalmark trial is evaluating the combination of amivantamab and capmatinib in unresectable metastatic NSCLC [19, 20]. The phase III MARIPOSA2 trial (NCT04988295) is evaluating amivantamab and lazertinib in patients who have progressed on osimertinib [21]. The ongoing CHRYSALIS trial (NCT02609776) has found that amivantamab monotherapy is less effective than combination therapy with lazertinib or chemotherapy [22]. A possible explanation is that combination therapy might better target different tumor subclones, reducing the risk of resistance than amivantamab.

Lastly, this case demonstrated a durable CNS response of 19 months in both the leptomeningeal and parenchymal lesions with amivantamab monotherapy. This finding challenges the established view that monoclonal antibodies with a high molecular weight have poor penetration through the blood-brain barrier (BBB) [23]. Approximately 25% of patients with EGFR-mutated NSCLC have CNS metastases at diagnosis, and the cumulative incidence of brain metastases is significantly higher in patients with EGFR mutated NSCLC [24]. Leptomeningeal metastasis (LM) is associated with a poor prognosis due to the aggressive nature of the disease and the difficulty of achieving effective drug concentrations in the CSF, even with treatment. The treatment options for LM in NSCLC includes surgery, radiation for symptomatic or bulky disease, systemic and intrathecal chemotherapy, and targeted molecular therapies, including EGFR and ALK inhibitors, alongside immunotherapy [25]. Despite the promise of third-generation TKIs, treatment of LM remains highly challenging due to limited BBB penetration [26, 27].

The CHRYSALIS trial suggested that a combination with lazertinib was more effective in achieving CNS penetration than amivantamab monotherapy, emphasizing the difficulty of BBB penetration for amivantamab [22]. In this case, the patient did not have CNS lesions before osimertinib treatment, yet follow-up scans revealed new CNS lesions after five months on osimertinib. Following initiation of amivantamab monotherapy, the patient achieved complete response in LMD, with six months of treatment restoring independent ambulation and daily function.

Most amivantamab trials, including CHRYSALIS and CHRYSALIS-2, have excluded or are excluding patients with untreated brain or other CNS metastases. A phase II trial (NCT04965090) evaluating the efficacy of amivantamab and lazertinib in NSCLC with progressive or new CNS metastases following prior treatment has included LMD, though it lacks an amivantamab monotherapy arm [28]. Currently, no established hypotheses or studies explain how amivantamab might penetrate the BBB. Further research is needed to explore this mechanism and better understand its effects on CNS metastases.

While rare, spontaneous tumor regression has been reported and should be considered as a factor [29–31]. However, in this context, it is unlikely to be the main cause of the patient’s response. The potential for a delayed immunological effect from previous immunotherapy is also acknowledged but is considered an unlikely primary contributor.

## CONCLUSIONS

In summary, we report the first case in which amivantamab monotherapy led to notably improved clinical outcomes in a patient with NSCLC harboring rare EGFR-mutated (G719A, A289V) with leptomeningeal carcinomatosis, following progression on osimertinib. Given the increasing prevalence of uncommon EGFR mutations and their association with poorer outcomes compared to more common EGFR mutations, it is essential to better understand these mutations and assess the efficacy of available treatments. Contrary to the expected poor BBB penetration of amivantamab, this case demonstrated a durable response in both the primary lesion and CNS lesions. The precise mechanisms by which amivantamab exerts its efficacy against rare EGFR mutations and crosses the BBB remain unclear and warrant further investigation. Future research into the effectiveness of amivantamab monotherapy for uncommon EGFR mutations and CNS metastasis, including LMD, could provide valuable insights to guide treatment strategies for similar NSCLC patient profiles.

## Abbreviations

ADLs: activities of daily living
ALK: anaplastic lymphoma kinase
BBB: Blood-Brain Barrier
CNS: Central nervous system
CNV: Copy number variant
CSF: cerebrospinal fluid
CT: Computed tomography
ctDNA: circulating tumor DNA
EGFR: Epithelial growth factor receptor
LM: Leptomeningeal metastasis
LMD: Leptomeningeal disease
MET: Mesenchymal-epithelial transition
mPFS: median progression-free survival
MRI: Magnetic resonance imaging
NGS: Next-generation sequencing
NSCLC: Non-small cell lung cancer
ORR: objective response rate
RECIST: Response Evaluation Criteria in Solid Tumors
SNV: Single-nucleotide variant
TKI: Tyrosine kinase inhibitor
VAF: variant allele fraction
